# Memory CD4^+^ T cells in chronic uveitis: pathogenicity, mechanisms, and therapeutic implications

**DOI:** 10.3389/fimmu.2026.1916577

**Published:** 2026-08-31

**Authors:** Maryam Shayan, Binapani Mahaling, Yihe Chen

**Affiliations:** Department of Ophthalmology, Schepens Eye Research Institute of Massachusetts Eye and Ear, Harvard Medical School, Boston, MA, United States

**Keywords:** chronic uveitis, IL-15, IL-23, IL-7, JAK-STAT, memory CD4+ T cells, non-infectious uveitis, ocular immune privilege

## Abstract

Chronic non-infectious uveitis (NIU) is a vision-threatening inflammatory disease in which relapses often occur despite corticosteroids, conventional immunosuppression, and biologic therapy. A growing body of experimental and human evidence indicates that disease persistence is not explained solely by transient effector inflammation. Instead, autoreactive memory CD4^+^ T cells can survive after the initiating trigger and retain retinal antigen responsiveness, sustaining persistent inflammation and reinitiating acute flares and exacerbations. In experimental chronic autoimmune uveitis, a specific subset of CD44^hi^IL-7R^+^IL-15R^+^ memory CD4^+^ T cells is maintained in the retina and peripheral secondary lymphoid organs (SLO), while additional autoreactive memory cells can persist in bone marrow niches through signal transducer and activator of transcription 3 (STAT3)-dependent mechanisms. Human studies further show expansion of memory and T helper 17 (Th17)-associated CD4^+^ T cell populations in NIU; however, the dominant immune cell subsets, clonotypes, and cytokine programs vary among heterogeneous clinical entities of NIU. This review summarizes how memory CD4^+^ T cells are generated, maintained, and linked to ocular tissue injury, with emphasis on their effector functions, IL-7 and IL-15-dependent survival signaling, STAT3-linked persistence, trafficking, and failed control by ocular immune regulation. We further discuss memory-directed therapeutic concepts grouped as disruption of survival/signaling, restraint of trafficking, and immune tolerization or restoration of ocular immune privilege. We propose the hypothesis that, in NIU subsets demonstrably sustained by pathogenic memory CD4^+^ T cells, durable remission may require elimination, restraint, or reprogramming of this reservoir rather than suppression of downstream cytokines alone.

## 1 Introduction

Uveitis refers to a heterogeneous group of intraocular inflammatory disorders involving the uvea and adjacent structures, including the retina, optic nerve, and sclera. It remains an important cause of visual morbidity, and more than one-third of affected patients develop measurable visual impairment. In developed countries, uveitis is among the leading preventable causes of blindness and has a disproportionate impact on working-age adults (1, 2). Chronic uveitis may be infectious or non-infectious and is commonly defined by persistent or recurrent intraocular inflammation lasting longer than three months (3). In non-infectious uveitis (NIU), immune dysregulation drives recurrent or sustained inflammation that can culminate in macular edema, retinal injury, fibrosis, optic nerve damage, and progressive visual loss (4, 5).

The therapeutic armamentarium for NIU includes systemic and local corticosteroids, conventional immunomodulatory therapy (IMT), including antimetabolites such as methotrexate and mycophenolate mofetil, calcineurin inhibitors such as cyclosporine and tacrolimus, and biologic agents including tumor necrosis factor-α (TNF-α) inhibitors (6). Nevertheless, treatment failure, relapse, and uveitic macular edema remain clinically important despite therapy (7–9). For example, in VISUAL II, treatment failure occurred in 39% of adalimumab-treated patients and 55% of placebo-treated patients during corticosteroid withdrawal (10). Similarly, the ADJUST trial showed that withdrawal of adalimumab after at least 1 year of controlled juvenile idiopathic arthritis (JIA)-associated uveitis resulted in treatment failure in 68% of patients switched to placebo versus 14% of those who continued adalimumab (hazard ratio 8.7), further illustrating the high risk of relapse after treatment withdrawal in a clinically defined uveitis subset (11). In addition, chronic uveitis often requires long-term or indefinite treatment, and prolonged systemic corticosteroid therapy carries substantial risk, including infection, adrenal suppression, osteoporosis, diabetes mellitus, Cushingoid changes, cataract, and glaucoma (12). Intraocular corticosteroids can similarly cause ocular hypertension, glaucoma, cataract, and local tissue complications (3). Conventional IMT can cause agent-specific cytopenias, hepatotoxicity, gastrointestinal intolerance, nephrotoxicity, hypertension, and reproductive toxicity, whereas TNF-α inhibitors are associated with serious infection, reactivation of latent tuberculosis or hepatitis, uncommon demyelinating events, and paradoxical inflammatory reactions (13, 14). These clinical limitations suggest that chronic uveitis represents a relapse-prone immune state rather than a short-lived inflammatory episode.

The eye is protected by immune privilege mechanisms that limit destructive inflammation and preserve visual function (15). The blood-retinal barrier (BRB), local transforming growth factor-β 2 (TGF-β2), α-melanocyte-stimulating hormone (α-MSH), programmed death ligand-1 (PD-L1), Fas ligand (FasL), retinoic acid, and tolerogenic antigen presentation by retinal pigment epithelium (RPE) and ocular antigen-presenting cells (APCs) create a microenvironment that favors immune restraint. These mechanisms do more than restrict immune cell entry; they influence T cell fate by promoting deletion, anergy, or regulatory differentiation of autoreactive lymphocytes that encounter ocular antigens (16–18).

Recent *in vivo* work reinforces the idea that ocular immune privilege is an active T cell fate checkpoint. In an adoptive-transfer model, naïve CD4^+^ T cells expressing a transgenic T cell receptor (TCR) specific for a retinal antigen were introduced into healthy recipients. After entering the eye, these antigen-specific cells were primed locally; a proportion differentiated into forkhead box P3-positive (Foxp3^+^) regulatory T cells (Treg), whereas the remaining cells acquired an anergic rather than a destructive effector program (19). This finding provides a mechanistic explanation for how the healthy ocular environment diverts autoreactive CD4^+^ T cells away from pathogenicity. However, this checkpoint can be circumvented. In a spontaneous autoimmune uveitis model, gut microbiota-dependent activation of retina-specific T cells generated IL-17A-producing, memory-like cells before clinical ocular disease, suggesting that pathogenic programming can begin outside the eye before local immune-privilege mechanisms are engaged (20).

In this review, we examine how memory CD4^+^ T cell fate decisions influence whether the eye maintains immune privilege or progresses toward chronic relapsing inflammation. We discuss mechanisms that normally impose tolerance, pathways through which those checkpoints are bypassed, and evidence that long-lived pathogenic memory CD4^+^ T cells contribute to disease persistence. We also evaluate strategies aimed at memory cell maintenance, JAK-STAT signaling, the IL-6/IL-23/IL-17 axis, trafficking, plasticity, and immune reprogramming rather than only downstream inflammatory cytokines.

## 2 Evidence for pathogenicity of memory CD4^+^ T cells in chronic uveitis

### 2.1 Preclinical evidence

CD4^+^ T cells have a central role in autoimmune uveitis, and evidence increasingly implicates the transition from acute effector responses to long-lived pathogenic memory CD4^+^ T cells as a key event in disease chronicity (21–23). Memory CD4^+^ T cells develop after antigen encounter, clonal expansion, contraction of the effector pool, and survival of antigen-experienced cells with enhanced recall capacity. They can be broadly classified into central memory, effector memory, and tissue-resident memory-like states, although these categories represent a continuum rather than rigid lineages (24). In chronic uveitis, this physiological memory program becomes harmful when retina-reactive CD4^+^ T cells adopt Th17, Th1, or mixed Th17/Th1-like effector phenotypes, resist deletion, persist in supportive niches, and repeatedly return to ocular tissue.

Experimental chronic autoimmune uveitis provides direct support for this concept. Chronic disease is associated with retinal and secondary lymphoid organs (SLO) accumulation of CD44^hi^IL-7R^+^IL-15R^+^CD4^+^ memory T cells that are capable of producing IL-17A, retain retinal antigen responsiveness, traffic back to the eye, and cause progressive structural and functional retinal injury (22, 23). Earlier work also identified bone marrow as a survival niche for interphotoreceptor retinoid-binding protein (IRBP)-specific autoreactive memory CD4^+^ T cells and showed that STAT3-dependent mechanisms support their localization or maintenance in this compartment. These cells can regain IL-17A- and interferon-γ (IFN-γ)-producing effector function after restimulation, linking memory persistence to recurrent inflammatory activity (21). Additional work demonstrated that IL-7 and IL-15 maintain pathogenic memory Th17 cells in autoimmunity, and more recent proof-of-concept studies showed that blockade of either pathway can deplete pathogenic memory CD4^+^ T cells and ameliorate chronic autoimmune uveitis (4, 25).

It remains unclear whether these retinal infiltrating memory cells are classified as circulating effector memory (T_EM_) or tissue-resident memory (T_RM_) cells. T_RM_ are defined primarily by durable non-recirculating behavior rather than by any single surface marker. CD69, CD103, CXCR6, and related markers are context-dependent, and CD4^+^ T_RM_ phenotypes can differ from those of the better-characterized CD8^+^ counterpart compartment. Accordingly, definitive assignment of retinal infiltrating cells as T_RM_ requires functional evidence of residence, such as parabiosis, intravascular labeling, tissue transplantation, or migration-blockade approaches (26–28).

### 2.2 Human evidence and disease heterogeneity

**Figure 1:**
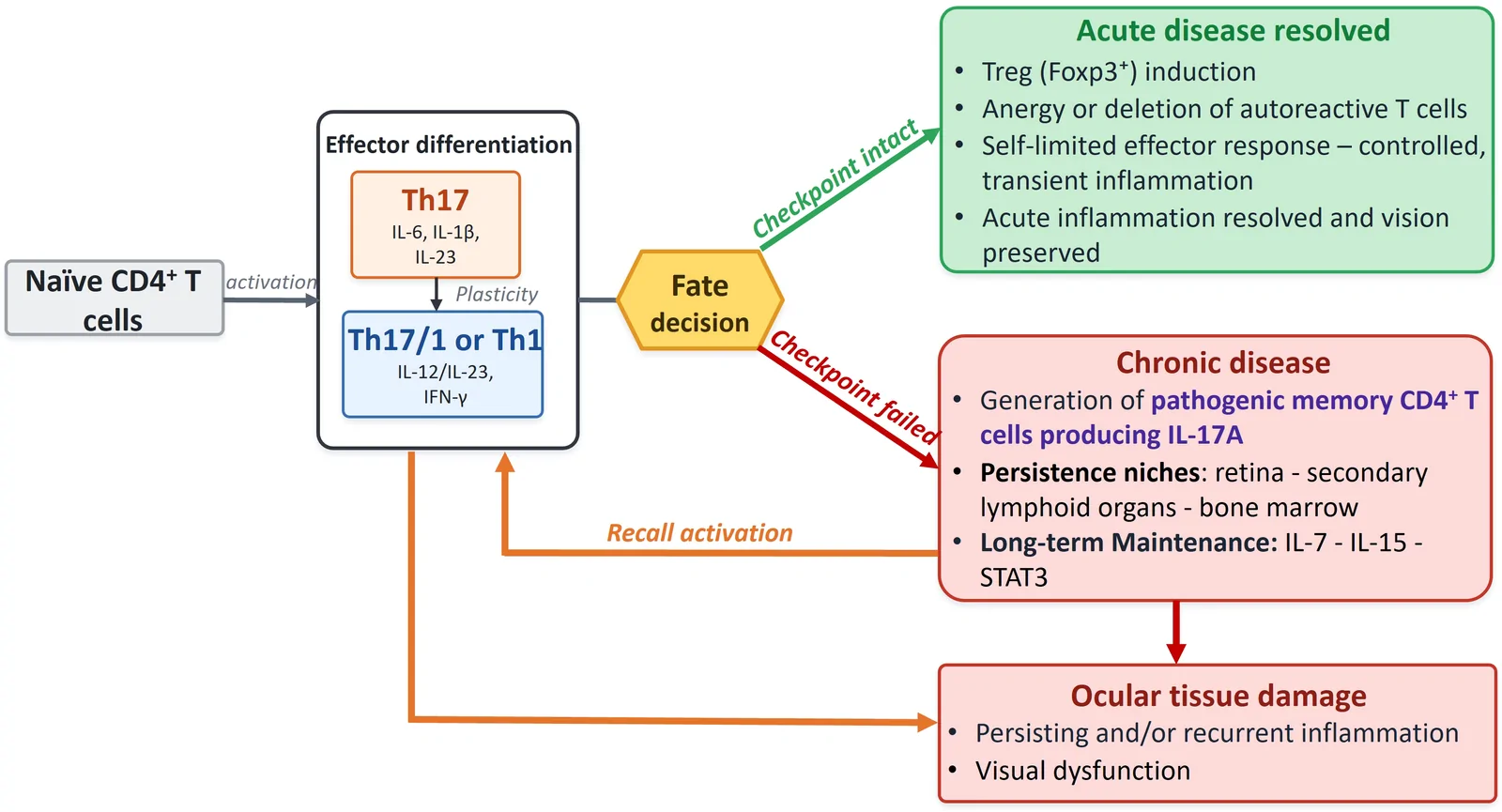
CD4^+^ T cell fate decisions in chronic uveitis. Retina-specific CD4^+^ T cells may be activated outside the eye, including at mucosal sites or within secondary lymphoid organs, and acquire Th17-like memory features before entering ocular tissue. In the healthy eye, immune-privilege mechanisms involving the blood–retinal barrier, retinal pigment epithelium, tolerogenic antigen-presenting cell, TGF-β2, α-MSH, PD-L1, FasL, and retinoic acid promote regulatory or anergic fates. When these checkpoints are bypassed or fail, autoreactive memory CD4^+^ T cells are generated and persist in ocular and systemic niches, where IL-7, IL-15, and STAT3-linked pathways support long-term maintenance. These memory cells can produce IL-17A and contribute to persistent retinal inflammation, while their recall activation promotes an exacerbated effector response and recurrent retinal injury.

Human studies are broadly consistent with this framework. Peripheral immunophenotyping in NIU, including Behçet disease and Vogt-Koyanagi-Harada (VKH) disease (29, 30), shows a shift from naïve toward memory CD4^+^ T cell populations, especially C-C chemokine receptor 6^+^ (CCR6^+^) and Th17-associated subsets, and higher abundance of these populations has been associated with subsequent need for systemic immunomodulatory therapy (31). In VKH disease-associated uveitis, increased IL-7 expression can amplify Th1 and Th17 responses, while IL-23 promotes IL-17A production by CD4^+^ T cells (32, 33). These observations are mechanistically relevant because IL-7 supports the persistence and responsiveness of memory CD4^+^ T cells, whereas IL-23 stabilizes and expands pathogenic Th17-lineage populations, linking active human uveitis to survival and lineage-maintenance circuits defined in experimental ocular autoimmunity (25, 32–34). Single-cell studies of ocular samples have shown clonal expansion of effector-memory CD4^+^ T cells with inflammatory signatures in VKH disease, whereas Behçet uveitis appears to have a stronger CD8^+^ cytotoxic program (35). Intermediate uveitis also shows local and systemic disturbance of the Th17/Treg axis (36). Together, these studies support a model in which chronic uveitis reflects both failure of ocular tolerance and persistence of autoreactive memory CD4^+^ T cell circuits (**Table 1**). This conceptual model is illustrated in **Figure 1**.

**Table 1:** Evidence linking memory CD4^+^ T cell pathogenicity to chronic non-infectious uveitis.

Evidence category		Key findings	Pathogenic implication	References
Preclinical model	Mouse chronic autoimmune uveitis	CD44^hi^IL-7R^+^IL-15R^+^ CD4^+^ memory T cells persist in retina and secondary lymphoid tissues.	Long-lived memory CD4^+^ T cells mediate sustained retinopathy.	(22, 23)
Preclinical model	IRBP-specific chronic uveitis model	Autoreactive memory CD4^+^ T cells reside in bone marrow through STAT3-dependent mechanisms.	Systemic survival niches preserve relapse-capable autoreactive cells.	(21)
Preclinical model	Chronic ocular surface autoimmune disease	IL-7 and IL-15 maintain pathogenic memory Th17 cells.	Homeostatic cytokines support pathogenic memory T cells.	(25)
Preclinical proof of concept	Mouse chronic autoimmune uveitis	IL-7 or IL-15 blockade reduces pathogenic memory CD4^+^ T cells and improves disease.	Memory maintenance is therapeutically targetable.	(4)
Human peripheral profiling	Non-infectious uveitis	CCR6^+^ and Th17-associated CD4^+^ T cell heterogeneity correlates with systemic immunomodulatory treatment.	Peripheral memory/Th17 profiles may stratify treatment need.	(31)
Human ocular single-cell profiling	VKH disease and Behçet uveitis	VKH disease shows clonal expansion of effector-memory CD4^+^ T cells; Behçet uveitis shows stronger CD8^+^ cytotoxic features.	Memory CD4^+^ T cell involvement is likely disease entity-specific.	(35)
Human cytokine axis	VKH disease	Increased IL-7 expression and IL-23-driven IL-17 production are observed in CD4^+^ T cells.	Human Th1/Th17 responses may be reinforced by memory-supportive cytokines.	(32, 33)

CCR6, C-C chemokine receptor 6; CD4, cluster of differentiation 4; CD8, cluster of differentiation 8; IL, interleukin; IL-7R, interleukin-7 receptor; IL-15R, interleukin-15 receptor; IRBP, interphotoreceptor retinoid-binding protein; NIU, non-infectious uveitis; STAT3, signal transducer and activator of transcription 3; Th17, T helper 17; VKH, Vogt–Koyanagi–Harada.

Taken together, current preclinical and clinical evidence indicates that memory CD4^+^ T cells are likely central in some NIU phenotypes and cooperative in others. Direct proof that antigen-specific tissue-resident or recirculating memory CD4^+^ T cells universally drive all chronic uveitis remains limited. Future studies should therefore define memory cell signatures, antigen specificity, tissue localization, and relapse relationships within disease-specific clinical contexts.

### 2.3 Tissue-resident memory CD4^+^ T cells beyond the eye

Work outside the eye shows that CD4^+^ T_RM_ cells can be either protective or pathogenic depending on tissue context and effector program. In mucosal infection, a local macrophage-derived chemokine network sustains protective CD4^+^ T_RM_ cells, whereas IL-2-dependent allergen-specific lung CD4^+^ T_RM_ cells can be sufficient to drive recurrent airway hyper-responsiveness (37, 38). These studies identify common themes relevant to uveitis: dependence on local cellular niches, rapid regional cytokine recall, and the capacity of a numerically limited resident population to organize broader inflammatory recruitment.

A particularly relevant comparison to NIU is progressive multiple sclerosis, another chronic autoimmune disease affecting an immune-specialized neural organ. CD4^+^ T_RM_ cells with inflammatory potential accumulate within central nervous system lesions, and combined targeting of resident and recirculating CD4^+^ compartments improves chronic disease in mice (39). This analogy supports, but does not prove, the possibility that ocular tissue-persistent CD4^+^ populations contribute to compartmentalized inflammation after control of circulating cells. It also emphasizes the need to distinguish true ocular residence from repeated recruitment and to determine whether resident and systemic reservoirs must be targeted together (26–28, 39).

## 3 Pathogenic mechanisms

### 3.1 Priming and memory formation

Memory CD4^+^ T cells arise after antigen recognition, costimulation, cytokine-directed differentiation, and survival of selected effector cells during contraction of the primary response. In protective immunity, this process allows rapid responses upon pathogen re-exposure. In autoimmune uveitis, it becomes pathogenic when retinal antigen-reactive CD4^+^ T cells escape deletion or regulatory deviation and differentiate into long-lived cells capable of repeated inflammatory recall.

Several factors favor this maladaptive transition during autoimmunity. The strength and duration of TCR stimulation, costimulation, the inflammatory cytokine milieu, and survival during contraction influence which activated CD4^+^ T cells enter the memory pool (40, 41). In ocular autoimmunity, pathogenic memory Th17 cells do not arise uniformly from one subset of effectors: T-bet^+^ RAR-related orphan receptor γt-positive (RORγt^+^) IL-17A/IFN-γ co-producing Th17/1 cells preferentially survive and seed the CD44^hi^ memory Th17 compartment, whereas conventional IL-17A^+^IFN-γ^−^ effectors contribute less efficiently (41). Persistent IL-23/IL-23 receptor signaling promotes conversion and expansion of these memory precursors, mechanistically linking acute inflammatory polarization to the formation of a memory pool (34, 40, 41).

Ocular tissue normally opposes this process. RPE cells can induce T cell anergy and support Treg activity, while ocular soluble mediators promote suppression and immune deviation (16–18). Experimental data show that the healthy eye can impose regulatory and anergic gene-expression programs on uveitogenic T cells (19). Thus, pathogenic memory formation in chronic uveitis likely requires local checkpoint failure, extraocular priming, or both.

Peripheral priming provides an important route to checkpoint bypass. Microbiota-dependent activation of retina-specific T cells before clinical disease indicates that autoreactive cells can acquire inflammatory and memory-like features before encountering the ocular microenvironment (20). After entering circulation, these cells may home to ocular tissue and initiate inflammation in a setting in which the BRB and local regulatory circuits are progressively weakened. This model helps explain how a highly immune-regulated tissue can still become a target of recurrent autoimmune injury.

### 3.2 Persistence circuits

Persistence is the feature that distinguishes chronic autoimmune memory from short-lived effector activation. In chronic autoimmune uveitis, autoreactive memory CD4^+^ T cells survive after the acute inflammatory phase and remain capable of driving sustained inflammatory retinal injury. These cells display a CD44^hi^IL-7R^+^IL-15R^+^ phenotype, retain responsiveness to retinal antigen, and can be detected in the retina and SLO months after disease induction (22).

IL-7 and IL-15 are central homeostatic cytokines for memory T cell survival. IL-7 supports anti-apoptotic signaling and homeostatic maintenance through IL-7R, while IL-15 contributes to memory cell persistence and recall competence (25, 40). Both IL-7R and IL-15R engage JAK-STAT signaling, including JAK1/JAK3 and STAT5, to support anti-apoptotic gene expression and homeostatic proliferation; IL-15 is frequently delivered by IL-15Rα-mediated trans-presentation, which can enhance proliferative and effector readiness (25, 42). In autoimmune ocular surface disease, both cytokines maintain pathogenic memory Th17 cells, and blockade of either pathway reduces memory Th17 survival and inflammatory activity (25). In experimental chronic uveitis, IL-7 or IL-15 blockade depleted pathogenic memory CD4^+^ T cells and improved disease parameters, demonstrating that the memory compartment is therapeutically actionable (4).

STAT3 is an additional persistence node. In chronic uveitis, IRBP-specific memory CD4^+^ T cells residing in bone marrow rely on STAT3-dependent mechanisms for their bone marrow localization or maintenance (21). This observation, together with the persistence of uveitogenic memory CD4^+^ T cells in SLO (22), indicates that pathogenic memory cells may be maintained not only in inflamed ocular tissue but also in systemic niches that protect them from local anti-inflammatory therapy and permit later reactivation. Bone marrow and SLO residency may provide stromal support, survival factors, and relative protection against deletion, creating a reservoir of autoreactive cells that can re-enter effector programs upon restimulation.

Collectively, IL-7, IL-15, STAT3 signaling, and specialized tissue niches form a persistence network that sustains a chronicity- and relapse-capable memory CD4^+^ T cell reservoir that can replenish target ocular tissues. Therapeutic failure in chronic uveitis may therefore reflect incomplete targeting of this reservoir rather than insufficient suppression of acute inflammation alone.

### 3.3 Persistent effector activity without overt recall

Pathogenic memory cells may maintain chronic inflammation even without a discrete antigen-rechallenge event. Effector-memory T cells have a lowered activation threshold and may receive continuous or intermittent signals from self-antigen, homeostatic cytokines, and the inflamed tissue milieu. In chronic ocular autoimmunity, memory Th17 cells can sustain IL-17A production under homeostatic and tissue-derived stimulation, and long-lived CD44^hi^IL-7R^+^IL-15R^+^ memory CD4^+^ T cells persist in the retina and SLO with retained inflammatory potential (14, 22). Thus, persistent low-level cytokine production by the maintained autoreactive pool may coexist with episodic recall responses and contribute to inflammation between clinically apparent flares.

### 3.4 Effector plasticity and ocular damage

During clinical exacerbations, memory CD4^+^ T cells are damaging because they combine longevity with rapid recall function. Compared with naïve T cells, memory cells require less stimulation to reactivate, produce cytokines more quickly, and traffic efficiently to inflamed tissues. In the retina, even limited recurrent inflammation can injure non-regenerating neural tissue and compromise visual function.

Th17-skewed memory CD4^+^ T cells are especially relevant to uveitis pathogenesis. IL-17A promotes chemokine production, leukocyte recruitment, and barrier dysfunction, while Th17 cells can acquire IFN-γ-producing Th17/Th1-like phenotypes. In chronic uveitis models, autoreactive memory CD4^+^ T cells can convert into IL-17A- and IFN-γ-co-producing effectors after restimulation (21, 22, 41). This plasticity may intensify tissue damage by combining neutrophil-recruiting and macrophage-activating inflammatory programs.

Activated pathogenic memory CD4^+^ T cells can injure the eye through multiple, mutually reinforcing pathways. They release cytokines such as IL-17A, IFN-γ, and granulocyte-macrophage colony-stimulating factor (GM-CSF), which activate retinal resident cells and endothelial cells and recruit myeloid cell infiltration. Activation of these cells increases chemokine and adhesion-molecule expression, promotes vascular permeability and BRB disruption, recruits additional leukocytes, and strengthens local antigen presentation, thereby establishing a feed-forward circuit that accelerates retinal tissue injury (22, 23, 43, 44). Repeated inflammatory activity can thereby transform episodic disease into chronic retinal remodeling, scarring, and functional decline.

The role of IL-17A is not linear. Although IL-17A-producing cells contribute to autoimmune uveitis, IL-17A can also limit Th17 pathogenicity through an autocrine IL-24-dependent negative-feedback pathway (45). Consistent with this complexity, secukinumab, an anti-IL-17A antibody, failed to meet its primary efficacy endpoints in three randomized controlled trials of NIU (46). Neutralizing IL-17A probably could not effectively deplete the autoreactive memory pool or prevent these cells from shifting toward IFN-γ, GM-CSF, IL-21, or other inflammatory outputs; moreover, removal of IL-17A may interrupt IL-24-mediated feedback that normally restrains Th17 pathogenicity (45, 46). Thus, the more durable therapeutic target may be the Th17-lineage memory compartment rather than a single terminal cytokine.

### 3.5 Tissue adaptation, immunometabolism, and remodeling

Tissue persistence requires adaptation to local nutrient availability, stress signals, and cell-cell interactions. Although much foundational T_RM_ biology derives from CD8^+^ cells, emerging CD4^+^ studies identify potentially targetable programs. Human and murine CD4^+^ T_RM_ cells can maintain preformed proinflammatory cytokine mRNAs in a translationally poised state regulated by the integrated stress response (ISR). Here, the “stress response” is a translational control pathway rather than T cell activation itself: at rest, phosphorylation of eIF2α suppresses global translation and promotes sequestration of cytokine transcripts in stress granules. Experimental T cell activation triggered eIF2α dephosphorylation, releasing this translational brake and permitting rapid production of IL-17A, IFN-γ, and GM-CSF. Conversely, genetic or pharmacological reinforcement of ISR–eIF2α signaling maintained the translational brake, reduced cytokine production, and ameliorated autoimmune kidney inflammation in mice. This mechanism is distinct from, but complementary to, immunometabolic adaptation: the ISR regulates translation in response to cellular stress, whereas nutrient-use pathways can support cell survival and persistence (47). In Crohn’s disease, fatty-acid oxidation supported apoptosis resistance and a proinflammatory CD4^+^ T_RM_ phenotype, demonstrating that metabolic adaptation can directly reinforce pathogenic persistence (48).

Direct evidence for comparable metabolic programs in ocular CD4^+^ T_RM_-like cells is not yet available. The retina nevertheless presents distinctive metabolic and stress conditions, and interactions with retinal pigment epithelium, Müller glia, microglia, endothelium, and infiltrating myeloid cells could shape survival and effector readiness. Recurrent cytokine and myeloid cell activation may also promote blood-retinal barrier remodeling, fibrosis, or angiogenic and neurotrophic growth factor programs. Whether memory CD4^+^ T cells directly produce such mediators or induce them in neighboring cells remains an open question that should be addressed with spatial transcriptomics, metabolomics, and cell-cell interaction analyses (26–28, 47, 48).

The mechanisms for the generation, maintenance, and trafficking of uveitogenic memory CD4^+^ T cells are summarized in **Table 2**.

**Table 2:** Mechanisms for the generation, maintenance, and trafficking of pathogenic memory CD4^+^ T cells in chronic non-infectious uveitis.

Mechanism	Main tissues, cells, molecules	Effect on memory CD4^+^ T cells	Expected ocular consequence	Therapeutic implication	References
Compromised ocular immune-privilege control	RPE, ocular APCs, TGF-β2, α-MSH, PD-L1, FasL, retinoic acid	Experimental RPE and *in vivo* eye models demonstrate induction of anergy and Treg programs; chronic uveitis is inferred to reflect incomplete or overridden local control.	When these programs are bypassed or insufficient, activated autoreactive cells retain pathogenic potential and are more prone to entering the memory pool.	Reinforce or restore tolerogenic ocular pathways rather than assuming complete checkpoint failure.	(16–19)
Peripheral priming	Gut microbiota, retina-specific T cells, IL-17	Activated memory-like autoreactive cells form before eye entry.	Ocular immune privilege is bypassed.	Target upstream priming or microbiota-linked activation.	(20)
Homeostatic survival	Retina, SLO, IL-7, IL-15, IL-7R, IL-15R	Survival and homeostatic proliferation are supported.	Durable memory pool persists.	Block IL-7 or IL-15 pathways.	(4, 25)
Tissue residency and metabolic adaptation	CD69, CD103, CXCR6, local myeloid/stromal niches, fatty-acid oxidation, integrated stress response	Supports tissue retention, survival, and rapid cytokine recall; markers and programs are tissue- and subset-dependent.	May sustain compartmentalized inflammation and remodeling after circulating inflammation is controlled.	Confirm bona fide residency and evaluate tissue-adaptive pathways without eroding protective local immunity.	(26–28, 37–39, 47, 48)
Survival niche localization	Bone marrow, stromal support, STAT3	Autoreactive memory cells are protected from deletion.	Systemic reservoir for relapse is maintained.	Target niche signals and STAT3-linked persistence.	(21)
Effector plasticity	Th17, Th1, IL-17, IFN-γ, IL-23	Memory cells convert to effectors during recall response.	Recurrent retinal inflammation and BRB disruption occur.	Target pathogenic recall and lineage plasticity.	(21, 33, 45)
Trafficking	S1P receptors, CCR6, CXCR3, adhesion molecules	S1PR1-dependent egress and CCR6/CCL20-directed Th17-biased homing enable recirculation and ocular entry.	Inflamed eye is repeatedly infiltrated.	Use trafficking restraint or chemokine-axis targeting.	(63–65, 67)
Feed-forward inflammation	Myeloid cells, retinal cells, cytokines, chemokines	IL-17A and IFN-γ from activated memory T cells activate retinal, endothelial, and myeloid cells, increasing chemokine production and antigen presentation.	Leukocyte recruitment, BRB permeability, and retinal tissue injury are amplified in a self-reinforcing circuit.	Combine memory targeting with local inflammation control.	(22, 23, 43, 44)

APC, antigen-presenting cell; BRB, blood-retinal barrier; CCL20, C-C chemokine ligand 20; CCR6, C-C chemokine receptor 6; CXCR3, C-X-C motif chemokine receptor 3; IFN-γ, interferon-γ; IL, interleukin; PD-L1, programmed death ligand-1; RPE, retinal pigment epithelium; S1P, sphingosine-1-phosphate; S1PR1, sphingosine-1-phosphate receptor 1; SLO, secondary lymphoid organs; STAT3, signal transducer and activator of transcription 3; TGF-β2, transforming growth factor-β2; Th, T helper; Treg, regulatory T cell; α-MSH, α-melanocyte-stimulating hormone.

## 4 Therapeutic implications

### 4.1 Targeting IL-7 and IL-15

The most direct memory-focused strategy is to disrupt survival signals that maintain pathogenic memory CD4^+^ T cells. IL-7 and IL-15 are currently the best-supported targets because they regulate memory cell persistence rather than only downstream cytokine secretion. In autoimmune ocular inflammation, IL-7 supports pathogenic memory Th17 cells (25). In chronic autoimmune uveitis, IL-7 blockade depleted pathogenic memory CD4^+^ T cells and improved disease activity, providing proof of concept that chronic disease can be treated by targeting the memory reservoir itself (4). Human VKH data showing increased IL-7 expression and IL-7-driven enhancement of Th1 and Th17 responses further support the translational relevance of this pathway (32).

IL-15 provides a related but mechanistically distinct maintenance signal. IL-7 is generally available as a soluble cytokine and signals through IL-7Rα and the common γ-chain, whereas IL-15 is commonly trans-presented by IL-15Rα on neighboring cells to the IL-2/15Rβ and common γ-chain receptor complex on T cells. These receptor and delivery differences may favor quiescent survival and homeostatic maintenance with IL-7 versus proliferation and effector readiness with IL-15, although the pathways overlap substantially (25, 42). Preclinical autoimmune studies show that IL-15 blockade can reduce the survival of pathogenic memory Th17 cells (25). In experimental chronic uveitis, IL-15 blockade depleted pathogenic memory CD4^+^ T cells and improved disease measures, similar to IL-7 blockade (4). These findings suggest that IL-7 and IL-15 are partially overlapping survival axes that sustain the pathogenic memory pool.

The therapeutic implication is that blocking one individual effector cytokine, such as IL-17A, TNF-α, or IFN-γ, may be insufficient if the underlying autoreactive memory compartment remains intact. By contrast, IL-7 or IL-15 blockade targets upstream maintenance signals. Translation to human disease will require careful dose selection, infection-risk monitoring, and biomarker-guided selection of patients whose disease is enriched for IL-7R^+^ or IL-15R^+^ memory CD4^+^ T cells.

There is currently no established human uveitis evidence for IL-7 or IL-15 blockade, and the benefit-risk profile therefore remains uncertain. Because these cytokines help maintain pathogen- and vaccine-specific memory, systemic blockade could impair antiviral recall, durability of vaccine responses, and immune surveillance (49, 50). Importantly, early human studies suggest that IL-7R blockade can be dose-titrated without uniformly eliminating protective immune compartments. In a phase Ib trial in adults with type 1 diabetes, RN168/PF-06342674 at 1–3 mg/kg preferentially reduced effector- and central-memory T cells while relatively preserving naïve T cells and Tregs; tetanus-toxoid recall responses were preserved at these doses but diminished at higher exposures, supporting a dose-dependent therapeutic window (51, 52). In a randomized first-in-human study in healthy volunteers, a single infusion of the anti-IL-7Rα antibody GSK2618960 achieved sustained receptor occupancy and inhibition of IL-7-mediated STAT5 signaling, without discernible changes in peripheral immune cell populations or serious treatment-related adverse events, although antidrug antibodies were common (53). Direct IL-7 neutralization has also entered early clinical development, with GSK3888130B evaluated in a phase 1 healthy volunteer study for multiple sclerosis (54). IL-15 neutralization also warrants attention to natural killer cells and CD8^+^ effector-memory cells: in rhesus macaques, anti-IL-15 caused profound natural killer-cell depletion, transient CD8^+^ effector-memory-cell loss, reduced tissue antiviral effector responses, and accelerated rhadinovirus reactivation, whereas treatment with the anti-IL-15 antibody AMG 714 did not reduce circulating human natural killer cells, underscoring species differences and residual uncertainty (55, 56). Early trials should therefore incorporate vaccination and infection history, baseline and serial lymphocyte subset monitoring, and time-limited dose escalation with active infection surveillance.

Local periocular or intravitreal delivery is conceptually attractive because it may reduce systemic immunologic exposure, but it could also compromise local antiviral surveillance and may not reach pathogenic cells maintained in secondary lymphoid organs or bone marrow. Conversely, systemic delivery may better access distributed reservoirs but carries greater risk to protective immune memory. Comparative pharmacokinetic and retinal-toxicity studies are therefore needed before choosing local, systemic, or staged delivery, and any ocular approach should test whether suppression within the eye is sufficient when extraocular reservoirs remain intact (4, 21, 22, 49, 50, 55, 56).

### 4.2 JAK-STAT pathway inhibition

IL-7 and IL-15 signal through JAK1/JAK3 and STAT5, whereas IL-6 and IL-23 engage related JAK-STAT modules, particularly STAT3. JAK inhibition may therefore attenuate several pathways that support memory cell survival, Th17 differentiation, and inflammatory cytokine signaling simultaneously (25, 42).

Clinical evidence in NIU remains preliminary. A recent review and a prospective cohort from the international AutoInflammatory Disease Alliance (AIDA) Network suggest that JAK inhibitors can reduce ocular inflammatory activity in refractory non-infectious inflammatory eye disease, but the available cohorts are heterogeneous and have not demonstrated selective depletion of pathogenic memory CD4^+^ T cells (42, 57). Potential advantages include oral administration and simultaneous pathway-level inhibition; limitations include infection and herpes zoster risk, cytopenias, lipid abnormalities, and class warnings for thromboembolic and major cardiovascular events. Future studies should determine whether clinical response is accompanied by reduced STAT3/STAT5 activation in IL-7R^+^ or IL-15R^+^ memory CD4^+^ T cells.

### 4.3 Targeting the IL-6/IL-23/IL-17 axis

IL-6, IL-23, and IL-17A act at distinct levels of the Th17 pathway. IL-6 promotes Th17 differentiation and can enhance memory CD4^+^ T cell recall, whereas IL-23 stabilizes and expands pathogenic Th17 and memory Th17 populations (33, 34, 58–60). In experimental autoimmune uveitis, IL-6 blockade reduces Th17 responses and disease severity (43, 44).

In human NIU, the STOP-Uveitis study of tocilizumab and the phase 2 SATURN study of sarilumab provided signals of clinical and anatomical benefit of blocking IL-6 receptor, particularly for uveitic macular edema, although neither trial was designed to test depletion of memory CD4^+^ T cells (61, 62). IL-23 targeting remains biologically compelling, but direct clinical evidence in uveitis is limited (60), whereas IL-17A blockade did not meet primary endpoints in randomized trials (46). These observations favor testing upstream or multi-node modulation with memory-focused pharmacodynamic readouts rather than assuming that neutralization of IL-17A alone removes the pathogenic memory compartment.

### 4.4 Trafficking restraint

Sphingosine-1-phosphate (S1P) is a bioactive lipid present at higher concentrations in blood and lymph than in SLO. Signaling through S1P receptor 1 (S1PR1) enables activated and memory lymphocytes to follow this gradient and exit SLO. Fingolimod, originally designated FTY720, is an oral S1P-receptor modulator that is phosphorylated *in vivo* and induces functional internalization of S1PR1, thereby retaining lymphocytes in SLO and reducing their entry into inflamed tissues (63–66). In experimental autoimmune uveoretinitis, fingolimod rapidly decreases ocular inflammatory infiltrates (63). Clinically relevant dosing also suppresses ocular inflammation, reduces cellular infiltration, and preserves the blood-ocular barrier (64), and FTY720 has shown suppressive effects in mouse experimental autoimmune uveitis (65).

This strategy is mechanistically relevant because circulating effector memory CD4^+^ T cells must enter the eye to execute recall inflammation. However, trafficking restraint does not necessarily eliminate the autoreactive memory reservoir. Relapse may recur if pathogenic cells remain viable and regain ocular access after treatment withdrawal. Systemic S1P modulation also carries safety considerations, including lymphopenia and ocular adverse events such as macular edema in other clinical settings. Thus, trafficking blockade may control tissue access but may need to be paired with memory depletion or reprogramming. Once a bona fide T_RM_ population is established, S1P-receptor modulation may have a limited effect on the resident pool, reinforcing the distinction between preventing new recruitment and eliminating established tissue residence (26–28, 39).

CCR6 provides a second, Th17-biased trafficking pathway. CCR6 is enriched on Th17 and selected memory CD4^+^ T cell subsets and directs migration toward C-C chemokine ligand 20 (CCL20). Human NIU contains expanded CCR6^+^ Th17-associated populations (31), and experimental ocular surface autoimmunity shows that CCL20 neutralization reduces Th17 recruitment and disease severity (67). Direct CCR6/CCL20 blockade has not yet been established in uveitis, but this axis warrants evaluation as both a biomarker and a candidate trafficking target.

### 4.5 Tolerization and immune reprogramming

Tolerization strategies aim to redirect autoreactive immunity rather than broadly suppress it. A recent preclinical study showed that combined anti-CD4 antibody and retinal antigen administration induced long-term disease control in autoimmune uveitis (68). This approach reduced ocular inflammation and supported durable immune modulation, suggesting that retinal antigen-specific tolerance can reshape the pathogenic CD4^+^ T cell response.

This concept aligns with ocular immune privilege. Healthy eyes promote regulatory and anergic T cell fates (19). Therapeutic tolerization may mimic or restore these programs by promoting Tregs, suppressing retinal antigen-specific recall, and limiting effector conversion of autoreactive memory CD4^+^ T cells. Such strategies may be particularly useful for relapsing disease, where durable immune recalibration is preferable to indefinite broad immunosuppression.

Endogenous immune privilege pathways may also be therapeutically useful. α-MSH and melanocortin signaling contribute to ocular immune regulation, and melanocortin 5 receptor (MC5R) expression has been linked to recovery of ocular immune privilege after uveitis (69). These findings support a broader strategy of restoring local regulatory circuits to constrain pathogenic memory responses.

### 4.6 Costimulation blockade in clinical uveitis

Few clinical therapies selectively target memory CD4^+^ T cells, but several interventions plausibly influence memory cell activation or function. Abatacept, a cytotoxic T lymphocyte-associated antigen 4 immunoglobulin (CTLA-4-Ig) fusion protein, blocks CD80/CD86-mediated costimulation and has been reported as a possible treatment for refractory juvenile idiopathic arthritis (JIA)-associated uveitis (70). Additional case reports and series support possible benefit in severe or refractory JIA-associated uveitis, although responses vary (71, 72).

Mechanistically, abatacept may reduce antigen-driven reactivation and expansion of autoreactive CD4^+^ T cells. However, established memory T cells often have lower costimulation requirements than naïve T cells. Costimulation blockade may therefore limit ongoing activation more effectively than it eliminates established memory reservoirs. Most clinical abatacept studies have not deeply profiled ocular or peripheral memory CD4^+^ T cell subsets before and after treatment, leaving the mechanistic effect on memory cells incompletely defined.

**Figure 2:**
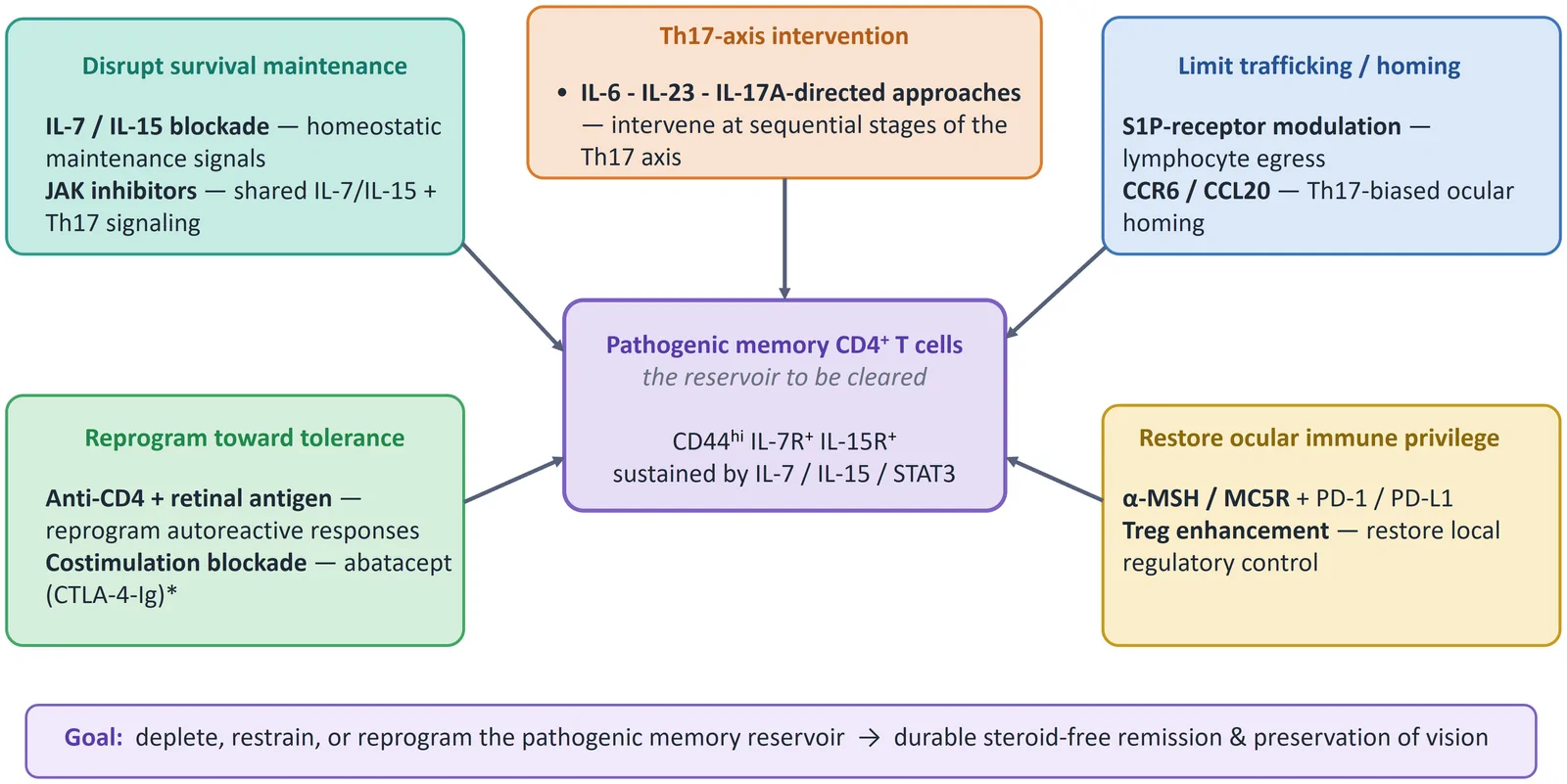
Therapeutic entry points for targeting pathogenic memory CD4^+^ T cells. Pathogenic memory CD4^+^ T cells may be targeted by disrupting maintenance, intercepting the Th17 effector axis, limiting tissue trafficking, inducing antigen-specific tolerance, or restoring ocular immune privilege pathways. IL-7 and IL-15 blockade directly target homeostatic survival and proliferative signals, and JAK inhibitors can suppress shared signaling downstream of IL-7/IL-15 and Th17-polarizing cytokines. IL-6-, IL-23-, and IL-17A-directed approaches intervene at sequential stages of the Th17 axis. S1P receptor modulation restrains lymphocyte egress, whereas CCR6/CCL20-directed strategies represent an additional candidate approach for limiting Th17 ocular homing. Anti-CD4 plus retinal antigen may reprogram autoreactive responses toward tolerance, and costimulation blockade with abatacept (CTLA-4-Ig) has been used clinically. α-MSH/MC5R and PD-1/PD-L1-linked pathways, together with Treg enhancement, may restore local regulatory control. Future trials should pair such interventions with memory-focused biomarkers and durable clinical endpoints. *Clinical use limited to case reports/series (JIA-associated uveitis), with variable response and incompletely defined effect on memory cells.

The therapeutic strategies targeting memory CD4^+^ T cell biology in chronic non-infectious uveitis outlined above are summarized in **Table 3**; **Figure 2**.

**Table 3:** Therapeutic strategies targeting memory CD4^+^ T cell biology in chronic non-infectious uveitis.

Strategy	Main target	Evidence level	Relevance to memory CD4^+^ T cells	Key limitation	References
IL-7 blockade	IL-7R-dependent memory cell maintenance	Preclinical chronic uveitis	Depletes pathogenic memory CD4^+^ T cells and improves chronic disease.	No established human uveitis evidence; possible impairment of antiviral memory, vaccine responsiveness, and infection control.	(4, 49, 50)
IL-15 blockade	IL-15R-mediated survival, trans-presentation, and homeostatic proliferation	Preclinical chronic uveitis	Reduces pathogenic memory CD4^+^ T cells and may preferentially limit proliferative and effector-ready memory states.	Potential effects on natural killer cells, CD8^+^ effector-memory cells, and antiviral surveillance; human effects may differ from animal models.	(4, 55, 66)
S1P receptor modulation	Memory cell trafficking	Preclinical uveitis	Restrains access of effector and memory cells to ocular tissue.	Does not necessarily eliminate the memory reservoir.	(63–65)
CCR6/CCL20-axis targeting	Th17-biased memory-cell trafficking	Preclinical ocular surface autoimmunity; biomarker evidence in NIU	May reduce recruitment of CCR6^+^ Th17-associated memory cells to ocular tissue.	No proof-of-concept therapy in uveitis; relevance is currently mechanistic.	(31, 67)
Anti-CD4 plus retinal antigen	Antigen-specific immune reprogramming	Preclinical autoimmune uveitis	May induce durable tolerance and reshape autoreactive CD4^+^ responses.	Translation and dosing strategy remain uncertain.	(68)
α-MSH/MC5R pathway modulation	Ocular immune privilege restoration	Preclinical/mechanistic	May restore the regulatory ocular microenvironment.	Unclear effects on modifying pathogenic memory CD4^+^ T cells.	(18, 69)
JAK-STAT inhibition	Shared IL-7/IL-15, IL-6, and related cytokine signaling	Early clinical cohorts and case-based NIU evidence	May simultaneously reduce memory cell survival signals, STAT3/STAT5 activation, and Th17 inflammatory programs.	Direct selective depletion of uveitogenic memory cells is unproven; systemic safety monitoring is required.	(42, 57)
IL-6/IL-23 axis modulation	Th17 differentiation, stabilization, expansion, and recall support	Preclinical uveitis plus clinical IL-6 trials; limited direct IL-23 clinical evidence	Targets upstream cytokines that maintain pathogenic Th17 states rather than only terminal IL-17A.	Optimal target, disease phenotype, and effect on memory CD4^+^ T cells remain uncertain.	(34, 60–62)
IL-17A blockade	Terminal Th17 cytokine	Clinical trials	Tests a downstream Th17 effector pathway.	Does not eliminate pathogenic Th17 lineage memory cells.	(45, 46)
Abatacept	CD80/CD86 costimulation	Clinical case reports and series in refractory JIA-associated uveitis	May reduce antigen-driven CD4^+^ T-cell reactivation.	Direct memory-cell effects are not well defined.	(70–72)

CCL20, C-C chemokine ligand 20; CCR6, C-C chemokine receptor 6; IL, interleukin; JAK, Janus kinase; JIA, juvenile idiopathic arthritis; MC5R, melanocortin 5 receptor; NIU, non-infectious uveitis; S1P, sphingosine-1-phosphate; STAT, signal transducer and activator of transcription; Th, T helper; α-MSH, α-melanocyte-stimulating hormone.

### 4.7 Trial design and biomarkers

Future uveitis trials should include immune endpoints that directly assess pathogenic memory CD4^+^ T cell biology and the pathways targeted by therapy. Candidate measures include CD45RO, CD44, IL-7R, IL-15R, CCR6, C-X-C motif chemokine receptor 3 (CXCR3), CCR7, CD69, CD103, T-bet, RORγt, IL-17A, IFN-γ, GM-CSF, phosphorylated STAT3/STAT5 after cytokine stimulation, and ocular or circulating IL-6, IL-23, and CCL20. TCR sequencing could determine whether expanded clones persist during remission and reappear during relapse. When feasible, antigen-specific assays using retinal peptides could help determine whether measured memory populations are autoreactive.

Clinical endpoints should also reflect memory biology. Short-term improvement in anterior chamber cells or vitreous haze is important, but memory-targeted therapy should also reduce relapse frequency, extend steroid-free remission, delay treatment failure, and preserve retinal structure and visual function. Optical coherence tomography (OCT), fundus imaging, fluorescein angiography, and electroretinography (ERG) could link immune changes with tissue protection.

A practical trial design would combine clinical outcomes with longitudinal peripheral blood immunophenotyping and, when available, ocular fluid single-cell profiling. Patients could be stratified according to memory/Th17 biomarkers such as CD45RO^+^IL-7R^+^CCR6^+^CD4^+^ T cell abundance, Th17/Th1 cytokine potential, or TCR clonality. For JAK-STAT- or cytokine-directed therapies, paired assessment of STAT3/STAT5 phosphorylation and IL-6/IL-23/CCL20 concentrations would provide target-specific pharmacodynamic evidence. A proposed biomarker and endpoint framework is provided in **Table 4**.

**Table 4:** Proposed biomarker framework for memory CD4^+^ T cell-targeted uveitis trials.

Domain	Candidate measures	Sample source	Rationale	References
Memory phenotype	CD45RO, CD44, CCR7, CD62L, CD69, CD103	Blood, ocular fluid when available	Distinguishes central, effector, and tissue-resident memory-like states.	(22, 24, 26–28, 35)
Survival signaling	IL-7R, IL-15R, BCL2, and cytokine-induced phosphorylation of STAT3/STAT5	Blood, ocular fluid when available	Tests whether therapy disrupts memory maintenance and shared JAK-STAT signaling circuits.	(4, 21, 25, 42)
Th17/Th1 plasticity	CCR6, CXCR3, RORγt, T-bet, IL-6, IL-23, IL-17A, IFN-γ, GM-CSF, and CCL20	Blood, ocular fluid when available	Captures Th17-lineage plasticity, cytokine support, and trafficking potential.	(31, 33, 34, 41, 67)
Clonality	TCR sequencing	Blood, ocular fluid when available	Identifies persistent or expanding autoreactive clones.	(35)
Antigen specificity	IRBP or retinal peptide stimulation where feasible	Blood, experimental tissue	Links phenotype to retinal antigen responsiveness.	(21, 22, 68)
Clinical durability	Relapse rate, steroid-free remission, time to treatment failure	Clinical data	Tests whether memory targeting produces a durable benefit.	(4, 7–9)
Tissue protection	OCT, fundus imaging, angiography, ERG	Ocular imaging and functional testing	Links immune modulation to retinal preservation.	(4, 22, 23, 64)
Pathway pharmacodynamics	Phosphorylated STAT3/STAT5 before and after IL-6, IL-7, IL-15, or IL-23 stimulation	Blood and ocular fluid cells when available	Confirms target engagement and distinguishes broad JAK-STAT suppression from selective cytokine blockade.	(42, 59)
Trafficking activity	CCR6/CCL20, S1PR1, and circulating-to-ocular clonotype overlap	Blood and ocular fluid when available	Tests whether therapy prevents pathogenic memory cell egress, homing, or ocular entry.	(31, 35, 66, 67)

BCL2, B-cell lymphoma 2; CCL20, C-C chemokine ligand 20; CCR, C-C chemokine receptor; CXCR3, C-X-C motif chemokine receptor 3; ERG, electroretinography; GM-CSF, granulocyte-macrophage colony-stimulating factor; IFN-γ, interferon-γ; IL, interleukin; IRBP, interphotoreceptor retinoid-binding protein; OCT, optical coherence tomography; RORγt, RAR-related orphan receptor γt; S1PR1, sphingosine-1-phosphate receptor 1; STAT, signal transducer and activator of transcription; TCR, T-cell receptor; Th, T helper.

## 5 Conclusion and future directions

Chronic NIU is heterogeneous; in biologically defined subsets, maladaptive memory CD4^+^ T cell circuits may be major drivers, whereas other entities may be dominated by CD8^+^ T cells, innate immunity, B cells, or mixed programs. Preclinical studies show that autoreactive memory CD4^+^ T cells can survive long after disease induction, retain retinal antigen responsiveness, traffic to ocular tissue, and mediate sustained retinal damage (22). These cells are maintained by IL-7, IL-15, STAT3-dependent programs, and supportive niches such as bone marrow and inflamed ocular tissue (4, 21, 22, 25). Human data support enrichment of memory and Th17-related CD4^+^ T cell populations in NIU, while also showing disease-specific differences across VKH disease, Behçet uveitis, and intermediate uveitis (31, 35, 36).

For NIU subsets in which a pathogenic memory CD4^+^ T cell reservoir is demonstrated, durable or treatment-free remission may require targeting that reservoir; this remains a biomarker-dependent hypothesis rather than a universal mechanism. IL-7 and IL-15 blockade provide the most direct preclinical evidence for depletion of uveitogenic memory CD4^+^ T cells. JAK inhibition and IL-6/IL-23-directed strategies may suppress interconnected memory and effector pathways, but their effects on the pathogenic memory reservoir require direct testing. Trafficking inhibitors may reduce ocular access, tolerization strategies may reprogram antigen-specific responses, and restoration of immune-privileged pathways may help re-establish regulatory control. Conversely, targeting terminal cytokines alone may leave autoreactive memory cells intact and able to switch inflammatory programs.

Several questions should guide future research. First, which human uveitis entities are primarily memory CD4^+^ T cell driven, and which are dominated by CD8^+^ T cells, innate immunity, B cells, or mixed inflammatory circuits? Second, are the relevant pathogenic memory cells tissue-resident in the eye, recirculating through blood and SLO, maintained in bone marrow, or distributed across compartments? Third, can IL-7R, IL-15R, CCR6, CXCR3, Th17/Th1 plasticity, or TCR clonality predict relapse or therapeutic response? Fourth, can memory-targeted therapy achieve steroid-free remission and eventually sustained treatment-free remission without compromising protective immune memory? Fifth, which metabolic programs and interactions with retinal pigment epithelium, Müller glia, microglia, endothelium, and stromal cells support ocular persistence and tissue remodeling, and do resident memory CD4^+^ T cells directly or indirectly induce profibrotic, angiogenic, or neurotrophic growth factor programs?

Addressing these questions will require longitudinal studies that integrate clinical phenotyping, ocular imaging, peripheral immune profiling, ocular fluid single-cell analysis, and antigen-specific assays. This approach can shift the field from broad immunosuppression toward mechanism-based immune restoration. If pathogenic memory CD4^+^ T cells are the reservoir that sustains relapse, therapies that delete, restrain, or reprogram this reservoir may offer a path toward durable remission and preservation of vision.
